# Supplementary material for: Splice-Junction-Based Mapping of Alternative Isoforms in the Human Proteome
Source: Cell Rep. Author manuscript; Available in PMC 2020 Jan 15. (PMC6961840; doi:10.1016/j.celrep.2019.11.026)
Supplement: 2 [file NIHMS1546469-supplement-2.zip › DF1/PXD010154/Liver/Liver_49_ALB_PHECYAKVLSVVLNQLCVLHEK.pdf]

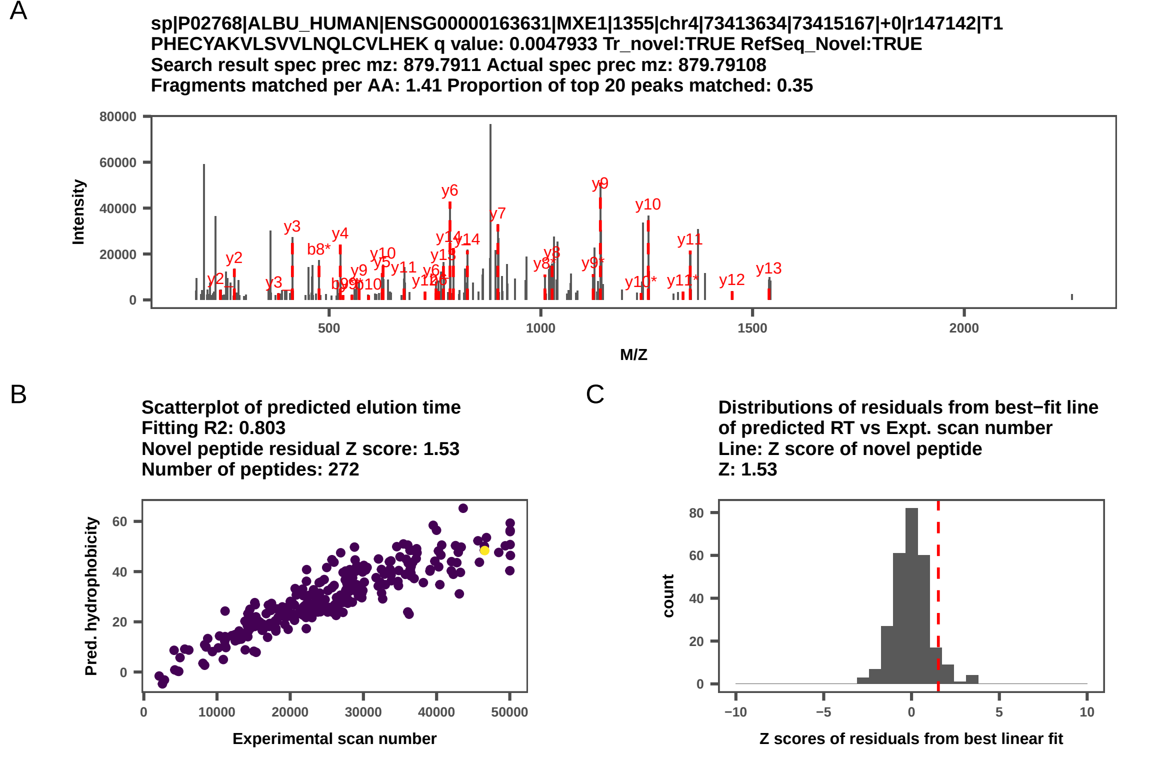
A
sp|P02768|ALBU_HUMAN|ENSG00000163631|MXE1|1355|chr4|73413634|73415167|+0|r147142|T1
PHECYAKVLSVVLNQLCVLHEK q value: 0.0047933 Tr_novel:TRUE RefSeq_Novel:TRUE
Search result spec prec mz: 879.7911 Actual spec prec mz: 879.79108
Fragments matched per AA: 1.41 Proportion of top 20 peaks matched: 0.35
Intensity
80000
60000
40000
20000
0
500
1000
1500
2000
M/Z
y2
y3
y4
b8*
y6
y7
y9
y10
y11
y12
y13
B
Scatterplot of predicted elution time
Fitting R2: 0.803
Novel peptide residual Z score: 1.53
Number of peptides: 272
Pred. hydrophobicity
60
40
20
0
0
10000
20000
30000
40000
50000
Experimental scan number
C
Distributions of residuals from best−fit line
of predicted RT vs Expt. scan number
Line: Z score of novel peptide
Z: 1.53
count
80
60
40
20
0
−10
−5
0
5
10
Z scores of residuals from best linear fit
